# Using Natural Language Prompts With AI Models for Low-Cost Assistive Software Design: Exploratory Comparative Evaluation

**DOI:** 10.2196/86786

**Published:** 2026-03-24

**Authors:** Francesc Antoni Bañuls-Lapuerta, Vicent Marti-Miralles, Rómulo Jacobo Gónzalez-García, Gabriel Martínez-Rico

**Affiliations:** 1 Campus Capacitas, Valencia Catholic University Saint Vincent Martyr, Carrer de Joaquin Navarro, 37, Burjassot, 46010, Spain, 96 363 74 12; 2 Doctorate School, Valencia Catholic University Saint Vincent Martyr, Valencia, Spain

**Keywords:** artificial intelligence, assistive products, digital accessibility, Gemini, ChatGPT, inclusive software

## Abstract

**Background:**

This study investigates the capacity of 7 artificial intelligence (AI) models, 5 free and 2 paid, to generate functional software for designing low-cost, personalized assistive products.

**Objective:**

The objective was to determine which models are most effective, accessible, and consistent in supporting nontechnical professionals in developing inclusive digital solutions and to assess the capabilities of commercially available and easy-to-access AI models to generate code from natural language interactions in the shape of a nontechnical assistive technology design process.

**Methods:**

Each AI model was prompted using natural language, without any technical input, to create a Python program that converts an arcade gamepad into an adapted mouse-like controller. Sixteen progressively complex functions were requested through standardized prompts, delivered without additional feedback or correction. Model performance was evaluated based on the number of successfully implemented functions and the average number of prompts required.

**Results:**

Paid models demonstrated markedly superior performance. Gemini Pro (Google) successfully implemented 14 of 16 requested functions with an average of 1.25 (SD 0.45) prompts, while ChatGPT Plus (GPT-5) achieved 11 functions with an average of 1.31 (SD 0.48) prompts. In contrast, free models produced between 0 and 4 functional outcomes, with DeepSeek and Gemini Free ranking the highest within their category. The enhanced outcomes of paid models were linked to improved contextual understanding, greater tolerance for natural language, and reduced conversational drift.

**Conclusions:**

Paid AI models, particularly Gemini Pro and ChatGPT Plus, exhibit strong potential as tools for bridging the gap between health or education professionals and software development. They enable the creation of affordable, user-centered assistive technology without requiring advanced programming skills. Nevertheless, human oversight and foundational literacy in prompt design remain crucial to guarantee functionality, reliability, and ethical use.

## Introduction

### Background

Artificial intelligence (AI) can be defined as the ability of nonhuman systems, machines, or software to simulate functions inherent to human intelligence, such as perceiving, reasoning, learning, planning, and anticipating future situations [1]. The recent advancements in the field have made clear that generative AI will open new scenarios in which human-machine collaboration will enable faster and more adaptive software solutions to meet the needs of people with disabilities [2]. In these contexts, the professional programmer assumes a supervisory role, while users without technical experience can create functional prototypes through intuitive AI-powered interfaces. It is thought that, by 2030, integrated assistants, such as the so-called HyperAssistant, will be capable of accompanying individuals throughout all stages of development, bridging the gap between software conception and implementation [3].

AI is transforming the way software is conceived and produced by allowing nontechnical users to participate actively in programming. Traditionally, this process was restricted to those mastering specific languages and methodologies; however, generative models in the shape of conversational AI systems have reduced the gap between natural language and coding, fostering digital inclusion and democratizing access to digital creation [4,5]. Generative assistants increase productivity and allow users with limited skills to build functional prototypes by using natural language–oriented programming, where users express requirements in everyday language and AI translates them into executable code, bridging human thought and computational structures. This AI integration in development environments additionally enhances efficiency through real-time suggestions and code autocompletion, while warning against technological overreliance [6-8].

### Design and Characteristics of Effective Prompts for Interaction With AI

The dialogue between a user and an AI system depends on how instructions or prompts, as they are known in the AI environment, are formulated. Prompts act as mediators between human intention and automatic language generation, shaping the trajectory of interaction, structuring intent, and bridging human goals with machine logic [9]. The first key element to achieving functional prompts is goal definition. Effective communication with conversational systems requires explicit purposes that limit and contain the semantic context. A prompt without a defined end, such as “tell me about economics,” produces generic responses, whereas “summarize in 200 words the effects of remote work on business productivity” provides focus and precision [10].

The second principle is linguistic specificity, referring to the fact that grammatically well-structured prompts enhance coherence. Since AI operates through probabilistic predictions, lexical clarity, thematic delimitation, and details about format or audience reduce ambiguity [11-13]. The internal structure should ideally include 5 essential elements: topic, style, tone, format, and context. It should also contain action verbs (such as analyze, compare, or synthesize) to guide specific cognitive-like operations [14,15]. This level of precision is fundamental, as prompt design directly influences the reasoning processes activated by the model [16].

Using these key principles and unifying different prompt explanations from the literature, we could divide prompts into 6 specific and differentiated types with their own goals (Table 1).

**Table 1.** Types of prompts used.

Prompt type	Goal	Example
Zero-shot or decision	Execute a task or produce a direct response without prior examples [17].	Explain the concept of neuroplasticity in simple terms.
Few-shot or iterative	Guide generation using prior examples [12,18].	Example 1: write a scientific abstract. Example 2: create a new one about artificial intelligence.
Chain of thought	Promote logical processes or explanations step-by-step [19].	Explain, step by step, how you arrive at the conclusion about the benefits of automation.
Sequential	Develop an idea or process step by step [11].	First, define the concept, then provide an example, and finally conclude.
Argumentative	Request a reasoned, well-supported position [11].	Argue why automation can be beneficial.
Verification	Review one’s own output and detect errors or biases [20].	Review your previous answer and indicate possible inaccuracies.

### Communicative Limitations of Nonexpert Users in Interactions With AI

Several studies agree that interactions between users and AI systems present communicative limitations stemming from the spontaneous and imprecise use of natural language. Users without technical training often formulate ambiguous prompts, making it difficult for the system to interpret communicative intent [21]. Additionally, the absence of nonverbal cues and the limited adaptability of artificial discourse exacerbate these differences, revealing that the effectiveness of interaction depends on the precision and structure of the language used [22].

From a qualitative perspective, users tend to treat chatbots as human interlocutors, reproducing communicative patterns based on empathy and reciprocity [23]. Users tend to apply Grice conversational maxims when evaluating virtual assistants. These maxims include quantity (providing the right amount of information), quality (saying only what is true and verifiable), manner (expressing oneself clearly and orderly, avoiding ambiguity), and relevance (keeping responses focused on the topic at hand) [24]. Complementarily, anthropomorphism, or trying to treat AI like a human, seems to induce a sort of relational dissonance that clearly shows a contradiction as users understand AI as nonhuman yet interact with it as if it were a human [25,26].

Generative models do not interpret implicit intentions but rather textual correlations; therefore, effectiveness depends on the specificity of the prompt [12,14]. Many users are unaware of this mechanism and rely on trial and error [27,28]. Furthermore, the absence of mental models about AI processing creates a cognitive gap between intention and response, reinforced by interfaces that simulate naturalness while not processing information cognitively naturally [29,30].

In summary, most users lack the metalinguistic skills required to design effective prompts, and bridging this gap demands a form of communicative literacy oriented toward AI.

### AI in Education: Barriers, Assistive Products, and Opportunities for Digital Inclusion

At this point, it is essential to analyze how AI can be integrated into educational and disability support contexts, expanding opportunities for student participation and learning. This link between technology and inclusion requires examining the barriers that still limit its effective use in classrooms. The analysis of possibilities for students with disabilities within the school setting must address the obstacles that hinder their access and active participation, emphasizing the importance of identifying factors that prevent the functional use of educational materials [31]. Students with disabilities face multiple barriers—including negative attitudes, lack of appropriate resources, and insufficient technological accessibility—which restrict their access to inclusive and equitable education [32].

Since the COVID-19 pandemic, information and communication technology has played a central role both in education and society [33]. However, the lack of technological accessibility remains a major barrier, as current learning materials heavily depend on digital tools. This not only affects curricular access but also the acquisition of digital skills essential for adulthood. Studies and hiring companies, such as iHire, highlight that 76% of people seek employment online, illustrating the importance of digital literacy for future labor inclusion [34,35].

To address these barriers, assistive products (APs) are key tools for ensuring the educational and social participation of students with disabilities. According to ISO 9999:2022, APs are products that optimize functioning and reduce disability, serving as intermediaries between personal abilities and environmental demands. The previous version of this standard (ISO 9999:2016) specified 3 key functions: facilitating participation, supporting or substituting body functions, and preventing limitations or restrictions in participation. APs associated with information and communication technology include both hardware and software, and the ISO 9999:2023 standard emphasizes their mixed nature, explicitly stating “including software” and recognizing their educational value within category e130 of the International Classification of Functioning [36-38].

Society and public administrations are responsible for ensuring that children with disabilities have access to the APs necessary for their personal and social development [39-41]. However, the World Health Organization warns that high costs and technical complexity limit access, particularly in educational contexts [42]. Although technological advances and globalization have partially reduced prices, technical barriers remain in specific contexts [43].

While many education and health professionals are familiar with physical APs, they often lack expertise in software-based solutions, which usually require technical support from IT specialists. This dependency increases costs and delays implementation. In this context, AI represents a major opportunity: its ability to generate and modify code accessibly allows the creation or adaptation of low-cost technological products, narrowing the gap between technical design and real student needs [44]. Although AI-generated software may not reach the technical refinement of professionally supervised development, it offers an effective, economical, and flexible alternative to enhance accessibility and educational participation for students with disabilities, particularly in low-resource or time-constrained contexts.

## Methods

### Overview

The software development process was carried out iteratively with the assistance of 8 different AI models, 6 of which were free and 2 were paid (Table 2). These models were selected to generate results applicable to contexts where cost may be a limiting factor. The chatbots were asked to program a software solution to convert an arcade fighting-style controller into a computer control device similar to an adapted mouse. Communication with the AIs was conducted using predetermined prompts prior to testing.

The objective was not only to obtain a functional program but also to significantly reduce the feedback provided to each AI and subsequently analyze each model’s ability to solve the problem as efficiently as possible, assessing whether it could produce a complete and functional solution. This approach enabled the comparison of performance, accuracy, and coherence across different platforms within the same experimental context, providing objective data on their real effectiveness in supporting software development through natural language instructions.

**Table 2.** AIs used, models, and price.

Company	AI^a^	Model	Price
OpenAI	ChatGPT (Free)	GPT-4.1 mini	N/A^b^
OpenAI	ChatGPT (Pro)	GPT-5	€23 (US $26.36)/month
Google	Gemini (Free)	Gemini 2.5 Pro	N/A
Google	Gemini (Paid)	Gemini 2.5 Pro	€21.99 (US $25.20)/month
Anthropic	Claude (Free)	Sonnet 4.5	N/A
DeepSeek	DeepSeek	DeepSeek V3.2	N/A
Microsoft	Copilot	o3 mini	N/A

a AI: artificial intelligence.

b N/A: not applicable.

To ensure experimental validity and avoid prior learning or contamination from conversational memory, each evaluation was conducted in entirely new conversations and, in the case of free plans, through newly created accounts. This strategy is grounded in empirical evidence showing that conversational history can induce bias or interference between tasks, thereby affecting model responses [45]. Similarly, literature on data contamination in language models indicates that any prior exposure to the evaluated information alters the reliability of results [46,47]. Even slight reformulations or lingering contextual information can modify the direction and quality of the generated output [48]. Therefore, initiating each session in a clean environment constitutes a methodologically necessary measure to control carryover bias, prevent contextual leakage, and ensure independence between trials, thereby preserving the external comparability of performance and accuracy across the evaluated models.

### Ethical Considerations

The Research Ethics Committee of Valencia Catholic University Saint Vincent Martyr approved the study (UCV/2023-2024/010).

### Test Protocol

The first step in initiating the coding process was to define the functions that could be beneficial and link each of them to the prompts that would be sent to the different AIs (Table 3). The definition of these prompts and the design of the conversation were based on two key concepts:

1. Iterative execution: the initial prompt is sent; the generated code is tested for functionality; and if it works successfully, the next prompt is sent with the intention of iteratively adding new features to the designed program.
2. Error handling: no feedback is provided, only the message “it doesn’t work, fix it.” This approach is based on the understanding that a person without technical qualifications might not comprehend the underlying problems in the code and would likely just ask the AI to fix the errors encountered. Such a person might be able to inform the AI that, for example, the joystick is not working but not describe more complex interaction failures or interpret Python error messages.

**Table 3.** Function desired and chosen prompt.

Function	Prompt
1. Move mouse with joystick	I have no knowledge of programming. I have a generic fighting style Game Pad that has a single joystick and several buttons. I need you to code a program in Python that allows the joystick on this device to generate the movements of the computer mouse.
2. Single right and left click	I need you to code left and click buttons like the mouse has.
3. Allow button reassignment	I need you to code in an app that allows the program to reassign keys in the gamepad.
4. Double left click from a single button	I need you to add an extra button that performs a double click in single click.
5. Toggle hold for left click	I need you to add an extra button that holds left click when pressed and releases when pressed again.
6. Scroll page up and down	I need you to add two extra buttons for scrolling up and down.
7. Increase and decrease volume	I need you to add extra buttons for volume up and down.
8. Assign a program to a button	I need you to add an extra button that opens Google Chrome when pressed.
9. Perform an automatic click after 3 seconds of mouse inactivity	Make it so that when the mouse automatically clicks once when standing still for 3 seconds.
10. Allow double-click with a configurable delay	I need you to add a delay that allows two clicks made within 3 seconds to register as a double click.
11. Type the user’s name with a button	I need you to add a button that writes the name Pelayo.
12. Pause and unpause	I need you to add a button that toggles between pause and play.
13. Ignore movement during the first second of joystick input	I need you to add a delay that ignores the first seconds of movement in any direction and then starts moving.
14. Automatically send a help email	I need you to add an extra button that automatically sends an email saying “Help” to francesc.banuls@ucv.es.
15. Generate an app that allows modifying sensitivity and delay time for automatic clicking	I need you to code in an app that allows me to change sensitivity of the joystick and how long the mouse waits after being still to automatically click.
16. Create a program and installer	I need you to compile the program made into an app by coding installer for this app.

Based on this premise, and to reduce inconsistencies, it was considered that modern AIs increasingly incorporate more integrated self-feedback mechanisms, independent from human input, and that models should be capable of performing such functions [49]. All testing was conducted by the authors, in the context of an autonomous reference center for disability in Valencia, Spain, during 4 days, from October 10, 2025, to October 14, 2025. Subsequently, all testing was conducted with the approval of the university’s ethics committee with code UCV/2023-2024/010.

Additionally, all testing was conducted using a Kubii USB Arcade Controller reference ODARCADE, connected via USB to a Windows 11 computer using Python 3.13 and having installed the libraries pygame and pyautogui.

The selection and design of the prompt constitute a key methodological element in this study, as its structure determines the comparative validity among the different AI models analyzed. Its uniformity follows the principle of instructional consistency, emphasizing that coherence in the formulation of commands is essential to ensure comparability between conversational systems [10]. Complementarily, the structure of the prompt conditions the type of cognitive processing activated by the model; therefore, keeping it constant allows the isolation of the intrinsic effect of each architecture [11]. Minimal wording differences can change both performance and the relative ranking of models, reinforcing the need to use a standardized prompt. Accordingly, the prompts were designed based on 3 theoretical principles [12,28,50].

The first is defining a nontechnical user role, representative of professionals without programming training. Contextualizing the interlocutor’s identity allows for adjusting response complexity, and that assigning an explicit role guides the model’s generation toward a semantically coherent and functional framework. This role also demands that all models are accessed from their web-found standard versions without adjusting models or using specific more technical or specially designed models for programming. This can be seen in the use of Copilot instead of specific instances coded into GitHub or other programming platforms [10,11].

The second is presenting a sequence of actions formulated in natural language and logical order. Procedural prompts activate step-by-step reasoning that promotes coherent and structured results, facilitating the translation of human descriptions into computational processes (as the de facto interaction mode with chatbots), although such prompts may carry a higher risk of drift [11].

The third is detailing functional requirements, such as cursor control or command execution. Precision in parameters or specific conditions reduces ambiguity and improves consistency of the output, making the prompt a cognitive interface between human language and automated execution [12,28].

These 3 concepts serve as guiding principles to mediate between the natural language of a nontechnical professional and the AIs. To more faithfully emulate the real process of software design by a person without advanced technical knowledge, 2 conditions were established to determine when to terminate the programming attempts.

The first one is that 3 consecutive nonfunctional generations either fail to execute, only partially implement, or lose communication with the gamepad. It is understood that if 3 consecutive iterations cannot fix inherited errors without feedback, it is unlikely that subsequent ones will do so.

Prompts are considered successful when they can correctly implement the full capabilities described in Table 1 for each prompt without compromising existing functionality. They are considered partially successful when they either maintain previous capabilities but only partially implement new functions or when they correctly implement new functions but lose previous ones. Finally, prompts are considered unsuccessful when they either fail to execute or lose communication with the gamepad. Partially successful prompts are marked as such to better understand the performance of different models but are functionally considered unsuccessful as they did not achieve the desired function. As such, 3 unsuccessful or partially successful prompts (with 2 tries each) stop interactions with a model.

The other way in which interactions with models are stopped is when reaching the daily prompt limit in free plans, to better reflect that the prompt limit is one of the most significant constraints of these models.

### Goals

The experimental design was developed with the explicit understanding that current AI systems are inherently nondeterministic and that the replication of results will be inconsistent [51]. Consequently, the study objectives reflect this fact:

- General goal 1: compare the effectiveness of the most widely used AI solutions as software programmers for creating personalized assistive products using nontechnical natural language.
  - Specific goal 1: describe the relationship between cost and effectiveness of the models used.
  - Specific goal 2: analyze the ease of use, consistency, and accuracy of each model.

## Results

### Overview

The 8 AI solutions initially proposed were used to progressively design robust code solutions, incorporating functions of increasing complexity inspired by APs with similar objectives.

The results can be divided according to the cost of the alternatives, distinguishing between those that are free of charge and those requiring a subscription. The latter provided more consistent results and better adaptation to the project’s functional requirements, demonstrating a greater understanding of the requested functions. However, it is important to note that these results should be interpreted within the framework of the specific characteristics of the study and that even under identical conditions, different outcomes could be obtained due to the nondeterministic nature of these technologies.

### Paid Alternatives

The subscription-based alternatives delivered consistent results, generating code that can generally be considered successful in meeting the required functions. This category includes ChatGPT Pro, based on the GPT-5 model, and Gemini Pro, which uses Google’s advanced Gemini Pro-2.5 model.

Regarding Gemini Pro, the AI solution developed by Google proved to be the most robust option. It successfully implemented 14 out of the 16 required functions within a single application, without any rollback of previously implemented features during the design process and generated functional code with an average of 1.25 (SD 0.45) prompts. Gemini was even partially able to anticipate the implementation of future functions in earlier items: item 2 was successfully integrated within item 1; item 6 was partially included in item 3 by adding a scrolling mode, although without the 2 specific keys intended for that function; and item 15 was partially addressed in item 3 through a joystick sensitivity slider. The only functions not achieved were items 14 and 16. Item 14 could be considered partially achieved, as the AI managed to open the email app and compose a message addressed to the correct recipient but was unable to send it. All partially achieved items are considered not achieved when counting successful items implemented. Regarding item 16, Gemini could not autonomously generate an installable app, and the alternative solutions proposed in Python were also nonfunctional.

One thing to note is that both items (14 and 16) did not work across the board for security reasons, as AIs are not capable of generating programs that have such deep access to your operative system. Seeing how the AIs approached a task they knew beforehand was impossible to fully be able to perform is interesting and shows the AIs’ problem-solving skill navigating natural language generated prompts, which may be difficult or impossible to fulfill.

Complementarily, OpenAI’s paid version (ChatGPT Pro using GPT-5) also demonstrated solid performance, successfully implementing 11 out of the 16 required functions and producing functional code with an average of 1.31 (SD 0.48) prompts. GPT-5 showed the same limitation as Gemini Pro with respect to sending the email in item 14 and was likewise unable to compile an executable file or provide stable Python-based compilation solutions. Additionally, OpenAI’s AI had difficulties implementing joystick controls in item 1, which caused the device to move the cursor only downward and to the right. This error persisted in subsequent iterations, affecting later results such as items 13 and 15, which were only partially achieved due to the lack of precise joystick control. ChatGPT also displayed inconsistency, producing multiple files for different functions instead of integrating them into a single app and rolling back previously implemented features without justification or explicit request.

### Free Alternatives

The free alternatives, in contrast to the subscription-based ones, produced notably less consistent results. In some cases, this was due to the limitations of the models themselves, which were unable to meet the functional requirements set for the task. In other cases, restrictions on the number of interactions significantly limited development, constituting a key distinction. It cannot be asserted that greater time investment would have yielded results equivalent to the paid versions, since the possibility of submitting additional prompts was not available. Therefore, in this study, the maximum number of interactions allowed by each model in its free version was established as a methodological limitation. Conversely, the models that failed to generate functional solutions can, for the time being, be considered inoperative for the intended function.

The free AI with the best performance was DeepSeek. This model generated the first 3 items in the initial prompt and even implemented item 2 without it being explicitly requested in item 1. However, the AI was unable to technically complete items 4, 5, and 6, implementing item 5 through a bind function that only allowed the assignment of a single key and caused the program to crash when attempting to add more combinations. This model generated functional items with an average of 1.88 (SD 0.34) prompts, achieving 4 out of the 16 required functions.

The second-best performance can be attributed to GPT Free. The free version of GPT successfully produced a functional prototype on the first attempt for the first 3 prompts but maintained the inconsistency observed in its paid counterpart. From the fourth prompt onward, it began producing nonfunctional code, leading to discontinuation after the seventh attempt. On average, it generated functional prototypes with 1.81 prompts and achieved 3 out of the 16 intended functions.

After GPT, in terms of performance, is Gemini Pro Free. Although it theoretically uses the same model as the paid version, its results differed appreciably. The free version produced correct and functional outputs up to the permitted prompt limit, forcing the process to stop prematurely. It successfully implemented the first 2 items and partially the third, generating a program that ran natively in the browser. Subsequently, time-based restrictions prevented continuation. On average, it produced functional prototypes with 1.88 prompts and achieved 2 out of the 16 complete functions.

The second-to-last worst-performing model is Claude. The Sonnet 4.5 model managed to implement only the first of the planned functions. After failing to execute functions 3, 4, and 5, its use was discontinued. Claude generated functional prototypes with an average of 1.94 (SD 0.25) prompts, achieving 1 out of the 16 proposed functions.

Finally, the individually worst-performing model is Copilot. Using its Deep Think model, Microsoft’s AI failed to generate any executable Python files within the first 3 prompts, leading to the termination of testing after the fourth attempt. It did not produce any functional prototypes, with an average of 2 prompts and a total of 0 out of 16 functions achieved.

**Figure 1.**
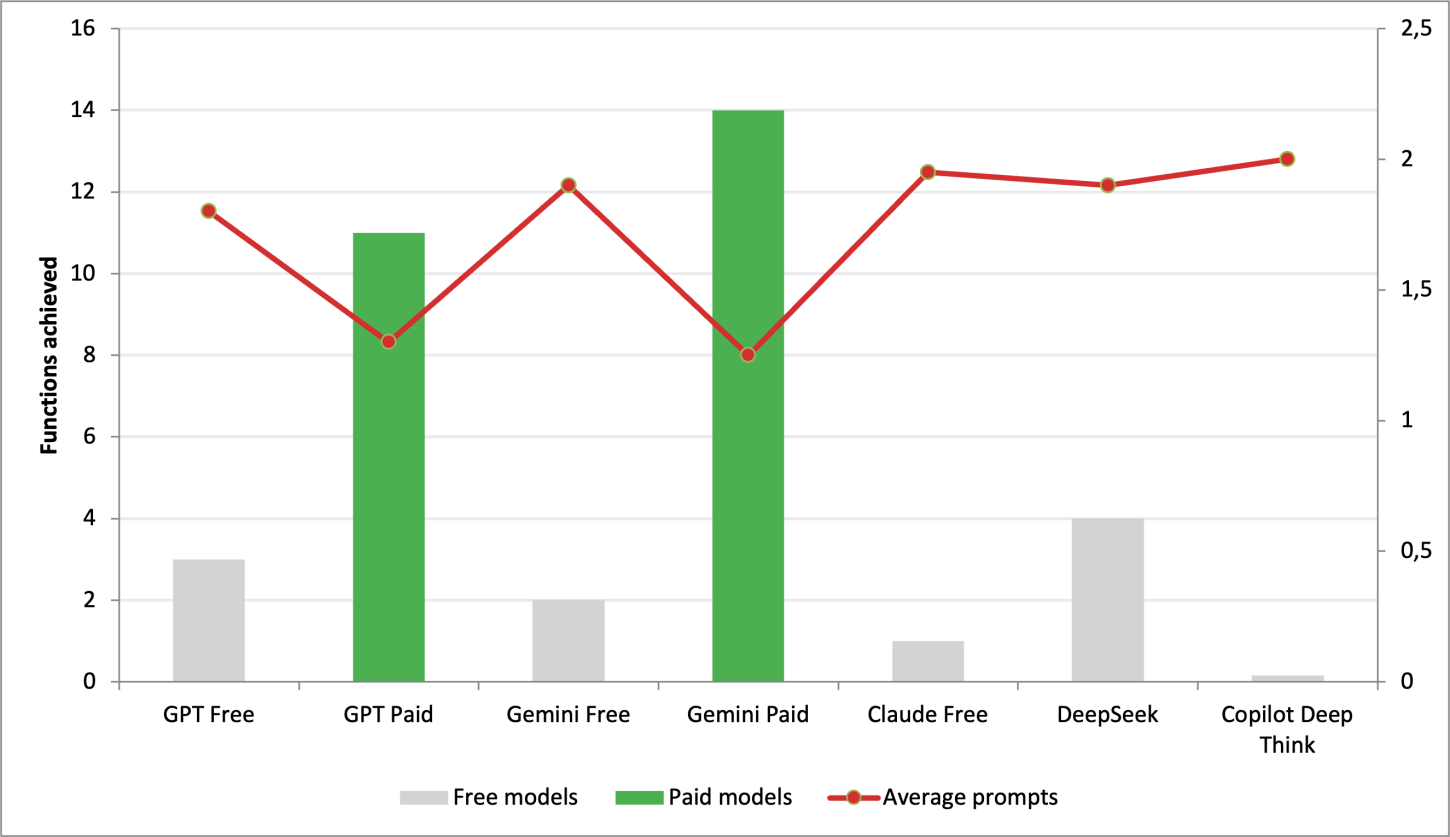
Prompts achieved and average prompts per model.

All models and a color-coded list of the items successfully implemented by each, along with the number of prompts required, are presented in Table 4. Additionally, prompts achieved and average prompts per model are presented in Figure 1.

**Table 4.** Functions achieved and prompts needed.

Required function	GPT Free	GPT Paid	Gemini Free	Gemini Paid	Claude Free	DeepSeek	Copilot Deep Think
1	✓^a^	×^b^	✓	✓	✓	✓	×
2	✓	✓	×	✓	×	✓^c^	×
3	✓	✓	✓^c^	×	×	✓	×
4	×	✓	×	✓	×	×	×
5	×	✓	×	✓	×	✓	×
6	×	✓	×	✓^c^	×	×	×
7	×	✓	×	✓	×	×	×
8	×	✓	×	✓	×	×	×
9	×	✓	×	✓	×	×	×
10	×	✓	×	×	×	×	×
11	×	✓	×	✓	×	×	×
12	×	✓	×	✓	×	×	×
13	×	×	×	✓	×	×	×
14	×	×	×	×	×	×	×
15	×	×	×	✓^c^	×	×	×
16	×	×	×	×	×	×	×

a Items achieved.

b Items not achieved or partially achieved. Partially achieved items are counted as unsuccessful.

c Functions already implemented or partially already implemented in previous interactions without direct prompts for their inclusion.

## Discussion

### Principal Findings

The results obtained show that the paid AI alternatives produced robust code, achieving 14 out of 16 functions with Gemini Pro and 11 out of 16 functions with ChatGPT Plus, respectively. The free-to-use alternatives, on the other hand, managed to integrate a variable, though consistently lower, number of functions ranging from none to 4 successfully implemented. The discussion of the results obtained can therefore be divided into 3 key areas (Figure 1).

### Superiority of Paid AIs

The use of paid AI systems offers several advantages, among which the most significant are access to more advanced models and the elimination or substantial expansion of the number of prompts allowed within a given period. These advantages naturally make paid AI solutions more reliable for performing complex tasks. The main improvements can be summarized in 3 key points.

Paid versions typically operate on newer, more advanced architectures with higher parameter capacity and improved optimization strategies and training based on larger and higher-quality datasets. In literature, larger and better-trained models consistently achieve superior results in both code generation and natural language understanding tasks. For instance, GPT-4 performs substantially better in programming tasks than earlier versions [52].

Due to these technical enhancements, paid models generally demonstrate a stronger grasp of natural language and communicative context, which increases the likelihood of successful interactions in this specific case scenario. They are also better at maintaining coherence across sequential instructions, retaining previously defined functions, and being better at avoiding contradictions and hallucinations. This allows them to integrate multiple features coherently and progressively with fewer rollbacks or loss of content [53-55].

The removal or significant reduction of restrictions on the number of prompts permitted per time interval is particularly relevant when considered alongside the highlighted advantages. Older free models often have weaker memory capabilities and greater difficulty minimizing inconsistencies, rollbacks, and errors across different interaction sessions [56]. Increasing the interaction limits from, for example, 5 per session in the free version of Gemini to 100, or from 10 every 5 hours in GPT-4.1 mini to 160 every 3 hours in GPT-5, not only eases time constraints but also reduces errors and can substantially improve code quality [57,58].

In summary, the differences in model capacity, contextual understanding, memory, and reasoning largely explain why paid AIs may achieve significantly more functional results than their free counterparts. This outcome is expected, as paying for a service naturally entails the expectation of better performance. However, another relevant point of discussion lies in comparing paid AIs with one another and questioning why Google’s AI seems to have outperformed GPT in this specific scenario, despite the latter’s longer development history and its recent update to version 5.

### Differences Between Gemini Pro and GPT-5

Although it is not possible to access the internal technical details of Gemini Pro, or of any major AI model, the existing literature published by the companies behind these models appears to support the results obtained in this specific case scenario, pointing to 3 key aspects that may explain why Gemini stood out as the most robust alternative for generating code from natural language given the restrictions and tasks asked.

On the one hand, Gemini may have better alignment between intention and execution. According to the Google Gemini team, the training of Gemini Pro placed greater emphasis on the correspondence between high-level instructions and structured code generation, giving it an advantage in translating complex requirements into functional code. The model achieved scores above 74% on specialized programming benchmarks [59].

This may also lead to much greater stability and coherence in long sessions. In tasks involving multiple functions (such as the 16 required in this study), the ability to maintain coherence across many iterations is crucial. If Gemini Pro manages internal session states more effectively, it becomes less prone to “forget” previously implemented functions or to inconsistently rename elements [60].

On the other hand, and especially relevant to the study, prompt optimization may be more tolerant of natural language. A distinct training approach regarding the model’s handling of natural language results in greater adaptability to intricate instructions, improved contextual understanding, and enhanced problem-solving abilities. This makes Gemini Pro more resilient to inaccuracies or ambiguities in prompts written in nontechnical natural language, whereas ChatGPT Plus may tend to require more carefully formulated instructions to produce reliable outcomes [59,60].

### Limitations and Strengths of Free AIs

As previously mentioned, free AI systems may present significant limitations related to the use of simpler and more restricted models, limited numbers of prompts, and, in some cases, the restriction to process multimodal information. These constraints make free models more challenging to use or less adaptable to users’ needs. The standardized subscription fee of US $20, as implemented by companies such as OpenAI and Google, remains possibly too expensive for low-resource contexts in many parts of the world.

In recent months, both companies have begun introducing country-based pricing, adjusting subscription costs to local purchasing power. OpenAI (2025) implemented multicurrency billing to reduce conversion costs, and in India, the Plus plan is now offered in rupees, alongside a lower-cost alternative named ChatGPT Go [61]. This strategy echoes *The Economist*’s Big Mac Index, which illustrates how multinational companies adjust prices according to economic context. AI can enhance accessibility in education, but its impact is constrained by the economic barriers preventing access to paid tools [62]. However, if AI design fails to account for income inequalities within a single country, despite global cost-adjustment strategies, it may reinforce digital exclusion. Thus, exploring the use and functionality of free versions and comparing them with paid alternatives as a potential source of social inequality becomes a social necessity to ensure equitable access to knowledge and accessibility [63,64].

In our test, among the free options, Gemini Free managed to implement 2 functions with relative stability during the first iteration, approaching a third before reaching the interaction limit. Considering the performance of its paid counterpart under fewer restrictions, it is likely that free versions could offer solid, no-cost solutions, albeit requiring a significant time investment, provided the model seems to be able to maintain internal coherence across sessions [59].

In contrast, GPT Free and DeepSeek achieved a greater number of items but lost track of the ongoing programming process, producing unstable code that would require the user to identify errors and explain them to the chatbot. While this may be possible, it is difficult to fully assess how much feedback a nontechnical user may be able to provide, and as such, it is difficult to give certain conclusions about these models. Professionals with intermediate technical knowledge could use these 2 models effectively to develop software solutions, but nontechnical users may be unable to [65,66].

Finally, Claude and Copilot, in their free versions, showed no real ability to generate operational code. In the case of Claude, this is understandable, since its website explicitly states that only the Pro version provides such capabilities [67]. The second model, Copilot, simply appears unable to generate functional, plug-and-play code. This is also due to the several factors, such as copilot being used in the web version (not optimized to do such tasks) without any specific model or implementation chosen [68]. In both case scenarios, using Claude Pro with programming capabilities and a version of Copilot optimized for programming may generate more competent results.

These findings may point to the fact that not all free versions are equivalent: some models are sufficiently robust for basic tasks, while others are less capable with the task chosen, the prompt limit, the version used, and many other factors contributing to their success.

One key limitation to have in mind is that only 1 run has been carried out with each model to mimic a real case scenario of a nontechnical user using these models. Given the nondeterministic nature of these models, more different runs around the same task may produce different results.

### Observations and Practical Implications

The observations, limitations, and implications for practice derived from this study can be summarized in 10 key points that integrate the main insights gained throughout the research. Four main points stand out as the implications of the results obtained.

Some AI models, such as ChatGPT (both 4.1 mini and 5), may produce inconsistent results, hallucinating or forgetting its progress. This can be seen by these models including renaming functions without apparent reason, changing project names, generating new programs instead of adding features to the existing one, or rolling back previously correct functions. This suggests that OpenAI models can generate the intended products but require a technical skill level beyond that of nonspecialist professionals, significantly complicating the process of obtaining functional prototypes for nontechnical users in this specific case scenario [66,69].

This behavior is sometimes categorized as AI “drift” and means that, during extended sessions, models may change behavior, forget previously defined functions, or misinterpret prompts that are conceptually equivalent. Maintaining a clear “narrative thread” and consistent prompt formulation is therefore essential. Natural language alone may not suffice, and the risk of accumulating errors that render the code nonfunctional remains high [70,71].

In this specific test, Gemini showed greater consistency interpreting natural-language instructions and required fewer technical reformulations or corrections. It also showed a lower risk of drift. In contexts where users represent the target population, these traits may reduce the barrier to entry and increase the likelihood of producing functional prototypes. The Pro version is notably more efficient and reliable, but the free version can achieve similar outcomes with sufficient time investment if the time between tries does not induce AI drift [72].

While AIs can generate code that is operational and useful, it must be remembered that its efficiency (execution time, memory usage, and design simplicity) is generally suboptimal. For this reason, professional supervision, or ideally full implementation, by a qualified software engineer remains preferable. Studies, such as EffiBench, have shown that AI-generated code tends to be, on average, less efficient than optimized human solutions [73].

The results obtained in this domain (low-cost AP adaptations with 16 functions with no feedback and in a single run) are constrained to the models and prompts used and to the absence of user feedback, maybe reflecting the condition of a nontechnical user. Other models or differently designed prompts might yield different outcomes. The practical implications are as follows:

1. For real-world AP adaptations: Collaboration with software professionals should always be prioritized when budgets allow. If it is not feasible, both paid models tested may be the most effective choice, as they perform better in programming tasks than other paid alternatives. When access to paid tools is possible, they remain the most robust option. However, free models such as Gemini Free, GPT Free, or even DeepSeek can provide functional results in exchange for greater time investment.
2. For low-budget or educational contexts: Free versions, particularly Gemini Free or GPT Free, can serve as valuable tools for initial prototyping, though additional iterations, revisions, and acceptance of functional limitations will be necessary [74].
3. Effective prompt design and problem segmentation: Breaking the task into smaller subfunctions and crafting clear, stepwise prompts improves the success rate, especially in free models.
4. Continuous monitoring and automated testing: Implementing automated unit tests (eg, by prompting the AI to generate its own test cases) may help detect errors or inconsistencies in the generated code.
5. Caution with long sessions or evolving prompts: Avoid overly long or evolving prompts within a single session and restart the interaction when the model shows signs of drift to preserve consistency.

### Conclusions

In conclusion, the comparative analysis of different AI models applied to the generation of functional software for designing personalized APs using natural language reveals clear differences in performance, usability, and consistency. Paid models, such as Gemini Pro and ChatGPT Plus, demonstrated greater efficiency and reliability, achieving 14 and 11 out of 16 required functions, respectively, while free alternatives ranged between 0 and 4. These results confirm that the technical advancements of newer and more sophisticated models, such as expanded context windows, improved multimodal communication, enhanced natural language comprehension, reduced drift, and fewer prompt limitations, directly influence the functional quality of the generated code.

Regarding the relationship between cost and effectiveness, Gemini Pro may stand out as the most balanced option, in this specific case scenario offering higher precision and coherence at a cost equivalent to other paid alternatives. For low-resource contexts, DeepSeek emerges as a viable free alternative that, despite its limitations, can produce acceptable results given sufficient time and technical supervision, while the free versions of Gemini can deliver solid outputs with adequate time investment.

In terms of ease of use and consistency, Gemini demonstrated greater tolerance for nontechnical natural language and more stable performance in long sessions, making it particularly suitable for users without programming knowledge, an essential aspect when the goal is to promote inclusive and low-cost design of APs.

Overall, the findings suggest that AI can serve as an effective bridge between health care or educational professionals and programming, enabling the creation of personalized assistive solutions without the need for advanced software development expertise. Nevertheless, the results also highlight the critical need for collaboration with qualified IT professionals, given the importance of human technical oversight, careful prompt design, and continuous testing to prevent drift and ensure the generation of stable software alternatives.

Future research could focus on exploring how AI models handle problem-solving from visual inputs (eg, images sent to chatbots), their capacity for self-generated prompt design, and the evaluation of emerging or alternative models.

## Abbreviations

AI: artificial intelligence
AP: assistive product
